# Supplementary figures and images for: Integrative multi-omics and network-based machine learning for early diagnosis of Parkinson’s disease
Source: PLoS One. 2026 Jan 6;21(1):e0329980. doi: 10.1371/journal.pone.0329980 (PMC12773809; doi:10.1371/journal.pone.0329980)

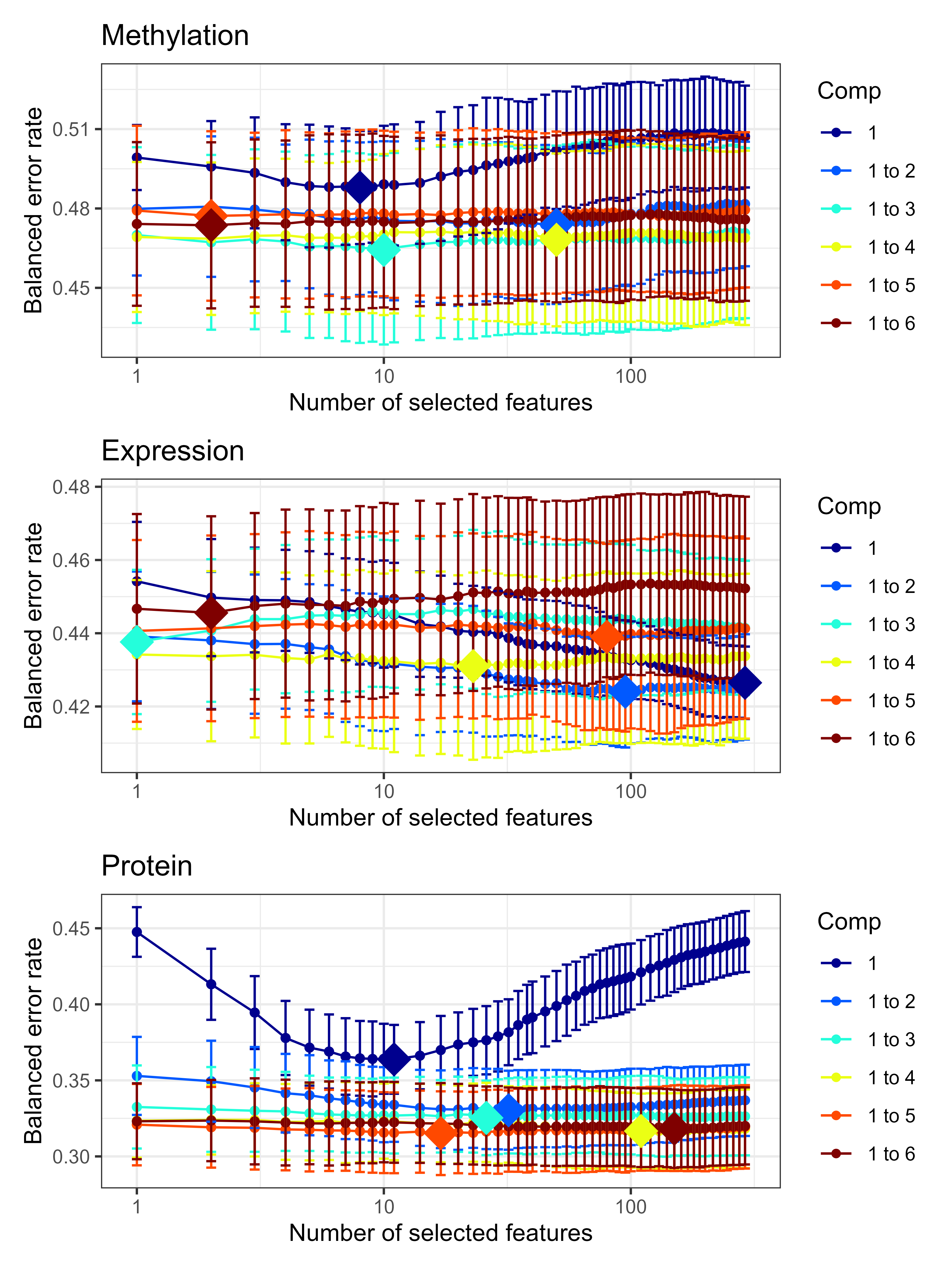

Supplement: S1 File — S1 Table. Bootstrap Stability Scores of Selected Features in sPLS-DA. S2 Table. Lists of the selected features in single- and multi-omics analysis. S3 Table. Results of function enrichment. S4 Table. The identified topological regulator. S5 Table. Table Stability Assessment of Network Topological analysis. S6 Table. Table Features of the train and test sets in machine learning. S7 Table. Table Features of the validation set in machine learning. S1 Fig. Balanced error rates (BER) of training models using sPLS-DA applied to DNA methylation, gene expression and proteomics datasets. A range of features from 3 to 300 (from 3 to 30 in steps of 3, from 30 to 60 in steps of 6, from 60 to 150 in the step of 15, and from 150 to 300 in the step of 30) was set for each component. Results were calculated with stratified 5-fold cross-validation and 100 random repeats. S2 Fig. (A)The performance of prediction models was evaluated under optimal parameter settings, with results measured by accuracy and AUC using stratified 5-fold cross-validation and 1,000 random repeats. The findings are presented as means ± standard deviation (SD). The notations MTEH, EXPR, PROT represent the outcomes of individual sPLS-DA analyses based on expression, methylation, and proteins respectively. The term MULTI denotes the results of an integrative sPLS-DA analysis across the three omics. (B) An overlapped features among three omics data across four distinct components identified through multi-omics integrative analysis. METH, methylation; EXPR, expression; PRO, proteins; MULTI, multi-omics. (ZIP) [file pone.0329980.s001.zip › Supporting information_600/S1 Figure.tif]

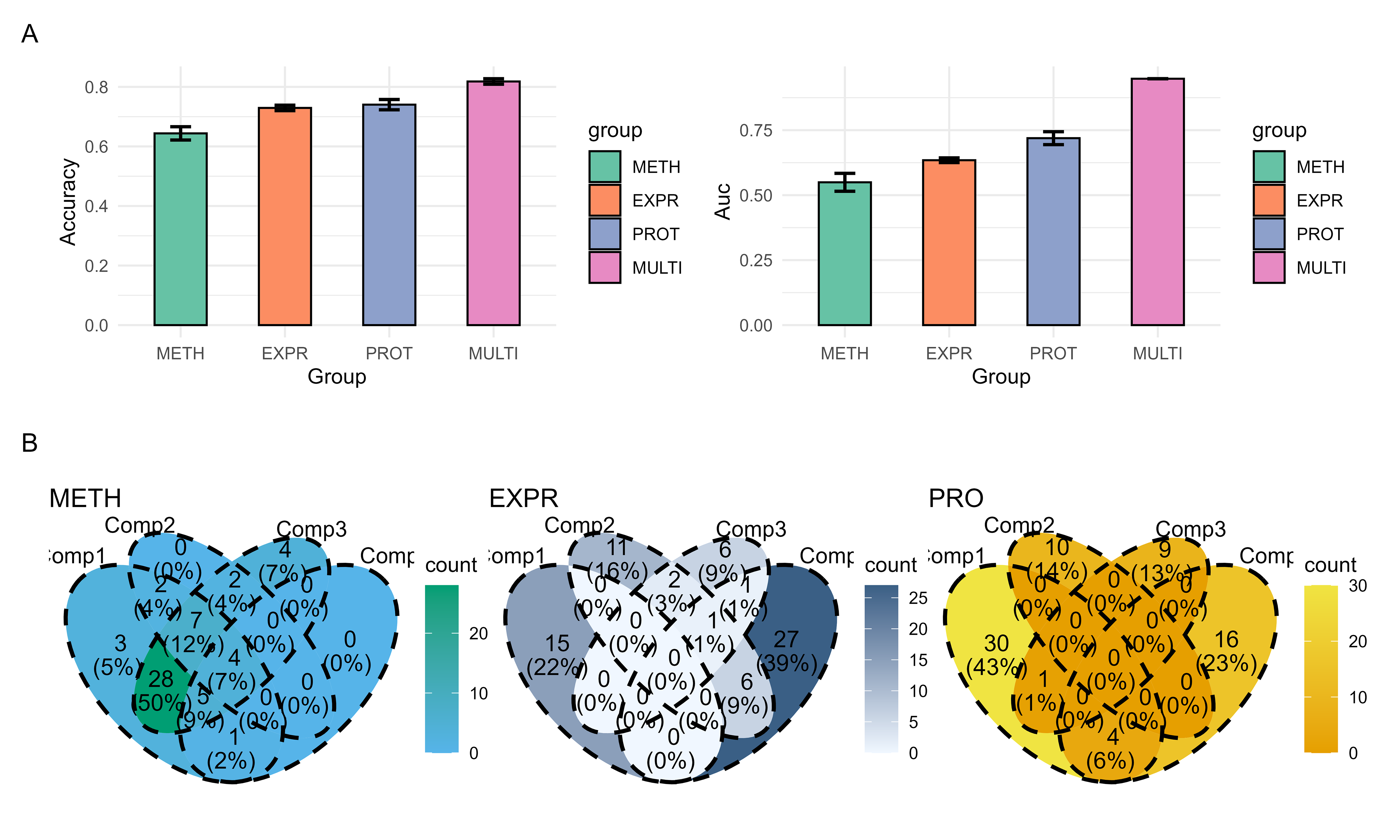

Supplement: S1 File — S1 Table. Bootstrap Stability Scores of Selected Features in sPLS-DA. S2 Table. Lists of the selected features in single- and multi-omics analysis. S3 Table. Results of function enrichment. S4 Table. The identified topological regulator. S5 Table. Table Stability Assessment of Network Topological analysis. S6 Table. Table Features of the train and test sets in machine learning. S7 Table. Table Features of the validation set in machine learning. S1 Fig. Balanced error rates (BER) of training models using sPLS-DA applied to DNA methylation, gene expression and proteomics datasets. A range of features from 3 to 300 (from 3 to 30 in steps of 3, from 30 to 60 in steps of 6, from 60 to 150 in the step of 15, and from 150 to 300 in the step of 30) was set for each component. Results were calculated with stratified 5-fold cross-validation and 100 random repeats. S2 Fig. (A)The performance of prediction models was evaluated under optimal parameter settings, with results measured by accuracy and AUC using stratified 5-fold cross-validation and 1,000 random repeats. The findings are presented as means ± standard deviation (SD). The notations MTEH, EXPR, PROT represent the outcomes of individual sPLS-DA analyses based on expression, methylation, and proteins respectively. The term MULTI denotes the results of an integrative sPLS-DA analysis across the three omics. (B) An overlapped features among three omics data across four distinct components identified through multi-omics integrative analysis. METH, methylation; EXPR, expression; PRO, proteins; MULTI, multi-omics. (ZIP) [file pone.0329980.s001.zip › Supporting information_600/S2 Figure.tif]
