# Supplementary material for: Integrative multi-omics and network-based machine learning for early diagnosis of Parkinson’s disease
Source: PLoS One. 2026 Jan 6;21(1):e0329980. doi: 10.1371/journal.pone.0329980 (PMC12773809; doi:10.1371/journal.pone.0329980)
Supplement: Attachment — Submitted filename: rebutter letter.docx [file pone.0329980.s003.docx]

**Reviewer 1**

**The study is limited to the PPMI cohort. Inclusion of at least one independent external dataset for validation is recommended to demonstrate the model’s generalizability.**

Thank you for the reviewer’s valuable comment regarding the generalizability of our findings. We agree that external validation is essential to assess the robustness and broader applicability of our model beyond the PPMI cohort.

In response, we have conducted external validation using independent datasets from the GEO database to evaluate the performance and transferability of our biomarkers. we identified an independent PD case-control dataset (GSE165083) with DNA methylation and expression profiles of whole blood samples which we preprocessed and normalized using methods consistent with PPMI. We applied the a priori defined feature panel (i.e., the features selected in the PPMI training model) to these external datasets without retraining. Classification was performed using the same XGBoost framework, and predictive performance was evaluated via accuracy, sensitivity, specificity and AUC, F1 scores.

The results show that our model achieves moderate performance in external cohorts: The TR Model obtained 51 genes, while the MO-TR Model garnered 139 features, including 28 CpG sites and 113 genes. Detail information on these features is showed in S5 Table. Performance metrics in the validation set indicated that the TR model exhibited the highest AUC, accuracy, and F1 scores (0.57, 0.62, 0.64, respectively), followed by the MO-TR model (0.56, 0.46, 0.59, respectively) and the MO model (0.54, 0.42, 0.44, respectively). Sensitivity was highest in the MO-TR model (0.83), trailed by the TR and MO models. However, specificity was lowest in the MR-TR model (0.14), succeeded by the MO and TR models (0.36, 0.50, respectively).

While differences in platformand cohort characteristics contribute to some performance drop compared to internal validation, the consistent signal reinforces the potential generalizability of our findings.

We acknowledge that full harmonization across studies remains challenging, and ideally more large-scale, multi-center omics datasets would be included. However, given current data availability, this external evaluation provides meaningful support for model transferability.

We have now incorporated these results into the revised manuscript: A new paragraph has been added to the section on materials and methods, PPMI and GEO datasets: data preprocessing (page 3). Additionally, the section on machine learning and model validation has been updated (page 6), and the section on results machine learning has been expanded (pages 12-13). Figure 6, which illustrates external validation performance, has been included. The limitations and implications of the study are now discussed in the section on Discussion (pages 16-17).

We sincerely appreciate the reviewer’s suggestion, which has significantly strengthened the rigor and credibility of our study.

**Consider discussing how the model might perform across different ethnicities and clinical settings.**

Thank you for the reviewer’s important and thoughtful comment. We agree that understanding the performance of our model across different ethnicities and clinical settings is critical for assessing its generalizability and equitable applicability in diverse populations.

While our model demonstrates robust performance in the PPMI cohort and shows promising transferability to external datasets, we acknowledge that its performance may vary across different ethnic groups and clinical contexts. The PPMI cohort is predominantly composed of individuals of European ancestry and recruited from specialized academic centers in North America and Europe.

Genetic and epigenetic biomarkers can exhibit population-specific variation due to ancestry-related differences in allele frequencies, methylation patterns, gene expression regulation, and environmental exposures. Additionally, clinical heterogeneity—such as variations in medication use, or diagnostic criteria across healthcare systems—may affect biomarker expression and model performance in real-world or resource-limited settings.

To date, most publicly available PD omics datasets (including those in GEO) share similar demographic limitations, which constrained our ability to perform a comprehensive cross-ethnic validation. However, we observed that several of our top biomarkers,such as *DNAJC6, SORL1,* and *NEDD4L.*

We emphasize that future validation studies must prioritize inclusion of diverse populations, including underrepresented racial and ethnic groups, as well as cohorts from varied clinical environments (e.g., community clinics vs. tertiary centers, different geographic regions). Only through such efforts can we ensure that precision medicine tools like ours are equitable and globally relevant.

We have now incorporated these results into the revised manuscript: a new paragraph added on page 16-17 of the Discussion section.

**It is unclear whether the identified biomarkers are stable across different random splits or perturbations of the dataset.**

We sincerely thank the reviewer for this insightful comment. We agree that assessing the stability of the identified biomarkers is crucial for evaluating their robustness and potential biological relevance.

To address this concern, we initially employed the PLS-DA algorithm, a linear and supervised method, to execute a feature pre-selection procedure for each individual Omic. This approach was designed to ensure robustness of the feature pre-selection step and mitigate the curse of dimensionality induced by high-dimensional data.

Secondly, we conducted the DIABLO methods to transform each single Omic dataset into latent components and maximize the sum of pairwise correlations between the latent components and a phenotype. It is able to identify features that are correlated across and within the Omics datasets. To improve PD prediction accuracy, it is critical to incorporate prediction uncertainty into our model for more accurate data modelling. We thus utilized multiple random splits of all individuals into training and testing datasets in a hold-out cross validation setting. Each split was performed in an unstratified way, not preserving the original proportion of PD cases and controls. In principle, this should yield a more conservative, generalizable model with stable biomarkers.

Lastly, we implemented the XGBoost algorithm, which integrates an in-built feature importance evaluation for feature assessment. The results obtained from this method were predominantly stable. Concurrently, we employed cross-validation with grid search to ensure the stability of the identified features. Feature standardization can mitigate technical variation and minimize the influence of non-biological factors during feature selection, thereby enhancing model stability.

The specifics were elaborated upon in the main text (Materials and Methods, Single- and Multi-omics Data Preprocessing and Integration Analysis, page 4; Machine Learning and Model Validation, pages 6-7).

**Recommend performing stability selection or bootstrap resampling to quantify feature selection reproducibility.**
Thank you for the reviewer's valuable comment. We agree that assessing the reproducibility of feature selection is crucial for ensuring the robustness and reliability of our results. We have performed extensive tuning of the sPLS-DA models (nrepeat = 100) to ensure the stability of our performance estimates. Furthermore, to directly quantify the reproducibility of feature selection as suggested by the reviewer, we conducted an additional bootstrap stability analysis (B = 1000). The results demonstrate high reproducibility in the selected features across data types in the sPLS-DA models. For the DNA methylation data, all selected features achieved a stability score above 0.8, indicating robust selection. In the gene expression data, 86.42% of the selected features surpassed a stability score of 0.8, and 99.48% exceeded a threshold of 0.6. Regarding protein data,  57.62% of selected features attained a stability score above 0.8, while 98.30% surpassed a threshold of 0.6.  These results confirm that the majority of features identified in our analysis are consistently selected across subsamples, supporting their reliability and robustness. The high stability scores, particularly for DNA methylation and gene expression features, strengthen confidence in the reproducibility of our feature selection process.

We have now incorporated these results into the revised manuscript: A new paragraph has been introduced in the section on Materials and Methods, specifically in the Parameter Optimization part, on pages 5-6. Additional information has also been provided in the Results section, under Single-omics Classification with sPLS-DA, on page 8. Furthermore, supplementary details have been included in Table S1.

**The study would be strengthened by experimental validation (e.g., qPCR, ELISA) for top-ranked biomarkers, particularly those with novel PD associations.**
Thank you for the reviewer’s thoughtful suggestion. We fully agree that experimental validation—such as qPCR or ELISA—of the top-ranked biomarkers would strengthen the biological interpretability and translational relevance of our findings, particularly for novel associations identified with Parkinson’s disease (PD).

While we recognize the importance of such validation, we regret that experimental follow-up is currently beyond the scope of this study due to resource and technical constraints. Our work is based on secondary analysis of publicly available omics datasets, and we lack access to matched biospecimens, laboratory infrastructure, and funding required for wet-lab validation at this stage.

Nevertheless, we have taken several steps to enhance confidence in the identified biomarkers: We applied rigorous cross-validation and bootstrap stability analysis to assess the robustness of feature selection. Top-ranked features show high reproducibility across resampling iterations (e.g., stability scores > 0.8 for key methylation and expression markers).

We contextualized our findings with existing literature, and several of the selected features are supported by prior evidence in neurodegeneration or PD-related pathways. Novel candidates have been highlighted with appropriate caution and are framed as hypotheses for future experimental testing.

We sincerely appreciate the reviewer’s point and fully acknowledge that functional or technical validation will be essential in future work. We have now explicitly stated this limitation in the revised manuscript (Section Discussion, page 16-17) and emphasized the need for experimental confirmation in follow-up studies.

**Highlight and prioritize a smaller subset of biomarkers with the highest translational potential.**

Thank you for the reviewer’s insightful suggestion. In response, we have revised our analysis to highlight and prioritize a more focused subset of biomarkers with the highest translational potential, rather than presenting an extensive list of candidates.

We cross-referenced the selected features with established Parkinson’s disease (PD) genetics and pathways. Notably, our analysis identified several known PD-associated genes, including DNAJC6, SORL1, and NEDD4L—all of which are supported by existing literature and have been discussed in detail in the revised Discussion section. For instance, DNAJC6 is implicated in synaptic vesicle endocytosis and has been linked to early-onset PD; SORL1 plays a role in APP trafficking and has emerging evidence in neurodegeneration; and NEDD4L, an E3 ubiquitin ligase, is involved in protein homeostasis pathways relevant to PD pathogenesis.These are addressed in the section discussion page 15.

Reviewer 2

**The analysis relies solely on a single cohort (PPMI) with no independent validation. To demonstrate clinical utility and generalizability, validation on an external dataset is highly recommended.**

Thank you for the reviewer’s valuable comment regarding the generalizability of our findings. We agree that external validation is essential to assess the robustness and broader applicability of our model beyond the PPMI cohort.

In response, we have conducted external validation using independent datasets from the GEO database to evaluate the performance and transferability of our biomarkers. we identified an independent PD case-control dataset (GSE165083) with DNA methylation and expression profiles of whole blood samples which we preprocessed and normalized using methods consistent with PPMI. We applied the a priori defined feature panel (i.e., the features selected in the PPMI training model) to these external datasets without retraining. Classification was performed using the same XGBoost framework, and predictive performance was evaluated via accuracy, sensitivity, specificity and AUC, F1 scores.

The results show that our model achieves moderate performance in external cohorts: The TR Model obtained 51 genes, while the MO-TR Model garnered 139 features, including 28 CpG sites and 113 genes. Detail information on these features is showed in S5 Table. Performance metrics in the validation set indicated that the TR model exhibited the highest AUC, accuracy, and F1 scores (0.57, 0.62, 0.64, respectively), followed by the MO-TR model (0.56, 0.46, 0.59, respectively) and the MO model (0.54, 0.42, 0.44, respectively). Sensitivity was highest in the MO-TR model (0.83), trailed by the TR and MO models. However, specificity was lowest in the MR-TR model (0.14), succeeded by the MO and TR models (0.36, 0.50, respectively).

While differences in platformand cohort characteristics contribute to some performance drop compared to internal validation, the consistent signal reinforces the potential generalizability of our findings.

We acknowledge that full harmonization across studies remains challenging, and ideally more large-scale, multi-center omics datasets would be included. However, given current data availability, this external evaluation provides meaningful support for model transferability.

We have now incorporated these results into the revised manuscript: A new paragraph has been added to the section on materials and methods, PPMI and GEO datasets: data preprocessing (page 3). Additionally, the section on machine learning and model validation has been updated (page 6), and the section on results machine learning has been expanded (pages 12-13). Figure 6, which illustrates external validation performance, has been included. The limitations and implications of the study are now discussed in the section on Discussion (pages 16-17).

We sincerely appreciate the reviewer’s suggestion, which has significantly strengthened the rigor and credibility of our study.

**Specificity of the models is low (~0.16), which limits practical application for early diagnosis. Further investigation into causes and improvements in this metric are needed.**

We sincerely thank the reviewer for highlighting this critical limitation regarding the low specificity (~0.16) of our model. We fully agree that high specificity is essential for any model intended for early diagnosis of Parkinson’s disease (PD), as low specificity would result in a high rate of false positives—posing significant risks for patient anxiety, unnecessary follow-up, and inefficient use of clinical resources.

Upon careful examination, we attribute this low specificity to several interrelated factors:

Standardization: The features before machine learning were not standardized, leading to poor performance of the model, especially with low specificity. After standardization, all models performed better than before, and the specificity increased compared to previously.

Class Imbalance in Early-Stage Data: The PPMI cohort used for training includes a relatively small number of healthy controls compared to PD patients, particularly in the early-stage subgroup analysis. This imbalance can bias classifiers toward predicting the majority class (PD), resulting in high sensitivity but low specificity. The results in validation set were superior than testing data.

Feature Specificity: While many selected features are involved in PD-relevant pathways (e.g., DNAJC6, SORL1), some may also play roles in general neurodegenerative or inflammatory processes, limiting their ability to uniquely discriminate PD from normal aging or other conditions.

Steps Taken to Address This Issue:

First, We have fully revised our preprocessing workflow to include standardization of all omics features prior to applying sPLS-DA. This step is essential given the heterogeneous scales and variances across different omics data types (e.g., methylation beta-values, log-transformed gene expression, normalized protein abundance). Second, we now explicitly report sensitivity, specificity, F1 scores, and AUC in a new supplementary table for transparency.

We have now incorporated these results into the revised manuscript:

A novel context has been introduced on page 6 in the "Materials and Methods" section, specifically in the "Machine Learning and Model Validation" subsection. Additionally, the results are comprehensively discussed in the "Results- Machine Learning" section on pages 12-13. The underlying reasons for these findings are further elucidated in the "Discussion" section on page 16.

**Some methodological details (parameter selection, handling of missing data,** **reproducibility of network analysis) require clarification or expansion.**
We thank the reviewer for highlighting the need for greater clarity in our methodology. We have now expanded the Methods section and added new supplementary materials to address the following points:

1. Parameter Selection in sPLS-DA and DIABLO Models

Model parameters were selected through a systematic, data-driven approach using repeated cross-validation to ensure robustness and minimize overfitting. The following is the original text of Parameter optimization.

Parameter optimization for sPLS-DA involved tuning two key elements: the number of components and the number of features per component(keepX). The component number was systematically evaluated from 1 to 6, based on empirical evidence that performance typically declines beyond five components. Feature selection was then optimized by exploring varying feature counts per component. For single-omics models (DNA methylation, gene expression, proteomics), the number of features ranged from 3 to 300, with incremental steps of 3 (3–30), 6 (30–60), 15 (60–150), and 30 (150–300). For integrative sPLS-DA (DIABLO), to ensure consistency across omics layers, feature numbers were uniformly varied from 10 to 50 in steps of 5, yielding nine candidate values per component per dataset. Additionally, a design matrix specifying inter-omics correlations was required. We defined the matrix with a correlation coefficient of 0.1 between omics layers, reflecting moderate biological connectivity.

Optimal parameters were selected based on model performance under stratified five-fold cross-validation with 100 random permutations. To account for class imbalance and ensure unbiased assessment, model performance was evaluated using the balanced error rate (BER). The optimal n comp was chosen as the smallest number achieving a plateau in performance.Final model validation was conducted using stratified five-fold cross-validation repeated 1,000 times, with predictive performance assessed via classification accuracy and receiver operating characteristic (ROC) curve analysis. Furthermore, to quantify the reproducibility of feature selection in sPLS-DA, we conducted a bootstrap stability analysis with 1,000 iterations (B = 1,000), computing selection frequencies for each feature across resampled datasets.The specifics were elaborated upon in the main text (Materials and Methods, Parameter Optimization, Page 4-5).

2. Handling of Missing Data

Missing values were handled in a data-type-specific and biologically informed manner:

DNA methylation：To refine the analysis, predictor variables exhibiting low variance across the samples were excluded; consequently, the top 10,000 most variable genes were selected for further investigation.

Gene expression：Genes that were either not expressed or expressed at low levels, defined as having an unnormalized median read count of less than 1 across all samples, were excluded from the analysis. These were considered likely to be non-informative features. In this dataset, predictor variables exhibiting low variance across all samples were excluded. Consequently, we focused on the top 5000 genes with the highest variability for subsequent analysis.

The specifics were elaborated upon in the main text (Materials and Methods, PPMI and GEO dataset: Data Preprocessing pages 3-4).

Sample handling

Since not all samples had complete multi-omics data, we included only those with complete measurements across all omics layers to minimize potential bias in downstream analyses, particularly in latent component estimation.

Following the exclusion of samples with incomplete data across all omics layers, a total of 305 participants were included from the PPMI cohort.

We have now incorporated these results into the revised manuscript: adding new content in the Methods section under "PPMI and GEO Datasets: Data Preprocessing" (page 3-4), and in the Results section under "Study Cohort and Data Refinement" (page 7).

Reproducibility and Stability of Network Analysis

This analysis comprehensively evaluates network stability through four interconnected pillars: Network robustness—the ability to maintain global connectivity under perturbation—was evaluated by simulating random node removal (5–50% of nodes in 5% increments, 20 simulations per step), with robustness defined as the ratio of the average size of the largest connected component post-removal to its original size. Node criticality was quantified based on the relative decrease in global efficiency (inverse average shortest path length) upon individual node deletion, identifying hubs essential for network integrity. Structural Stability is characterized by key topological metrics: average path length (information propagation efficiency), clustering coefficient (local modularity), modularity (strength of community structure, with Q>0.4 indicating strong modularity), and degree/betweenness centralization (highlighting the concentration of connections and mediation influence, revealing hierarchical organization and small-world properties). Finally, Regulatory Node Stability specifically examines the topological role of biological regulators, we computed the ratio of average degree between regulatory and non-regulatory nodes (regulator degree ratio) and their average betweenness centrality; a ratio >1 indicates that regulators are more highly connected and centrally positioned within the network, with underscoring their potential role as central hubs and bridges coordinating network activity.

The results revealed that the topological regulatory network exhibits small-world properties (average path length = 3.03, clustering coefficient = 0.67) and a high degree of modularity (Q = 0.56), indicating functionally segregated communities with efficient information transfer. The network showed moderate robustness (score = 0.59) and significant centralization (degree centrality = 0.41), with a subset of highly connected hub nodes. Notably, regulatory factors exhibited higher connectivity than their targets (ratio = 1.18), suggesting their central role in coordinating molecular activity. These topological features support the biological relevance and structural stability of the inferred network.

We have now incorporated these results into the revised manuscript: adding new content in the Methods section under "Network Topological Analysis" (page5-6), and in the Results section under the same heading (pages 11–12)

**The chosen cross-omics correlation coefficient (0.1) is weak. This choice requires either justification with supporting analyses or tuning for optimum model performance.**

We thank the reviewer for this insightful comment. The design matrix parameter in DIABLO controls the strength of inter-omics correlations enforced during component extraction, and its choice indeed influences model sparsity and performance.

While 0.1 may appear weak in absolute terms, it is consistent with empirical observations in multi-omics studies, where biological signals across different molecular layers (e.g., transcriptome, proteome, metabolome) are often modest due to biological complexity, technical noise, and post-transcriptional regulation. For instance, a study such as Jin H et al.,Clin Transl Med. 2021 had reported inter-omics correlations of 0.1 in integrative analyses of intraplaque hemorrhage-associated cardiovascular signature. A correlation of 0.1 reflects a moderate level of biological connectivity while maintaining model stability and avoiding overfitting, especially in high-dimensional settings. In multi-omics data (e.g., transcriptome, proteome), biological signals are often subtle due to post-transcriptional regulation, protein turnover, and technical variability. A stricter threshold would result in highly fragmented networks with isolated nodes, limiting integrative insights.Therefore, we believe that 0.1 is a biologically plausible and conservative estimate that balances sensitivity and specificity in detecting meaningful cross-omics associations.

We have now incorporated these results into the revised manuscript:

Added a new context in section discussion, page 14

**Statistical reporting (confidence intervals, repeated splits, class balancing) should be strengthened.**

We thank the reviewer for this valuable suggestion. We agree that robust statistical reporting is essential for evaluating model performance. In response, we have strengthened our statistical reporting in the following ways:

Confidence intervals: Model performance metrics—including accuracy, sensitivity, specificity, F1 scores and AUC—are now reported with 95% confidence intervals, calculated across 1,000 iterations of stratified 5-fold cross-validation. These CIs reflect the variability due to data splitting and sampling. The results of 95% CI and mean now are presented in table 3 and table 4.

Repeated cross-validation: To ensure stable and generalizable estimates, we used stratified 5-fold cross-validation repeated 1,000 times. This approach reduces variance from random partitioning and provides more reliable performance estimates than a single CV split. Detailed methodological descriptions are provided in the "Machine Learning and model validation" subsection of the Materials and Methods section in the main text (pp. 6–7).

Class imbalance handling: Given the potential for class imbalance in our dataset, we adopted multiple strategies: Balanced Error Rate (BER) was used during parameter tuning to optimize model performance across both classes; All cross-validation folds were stratified to preserve class distribution in training and test sets; For final evaluation, we report sensitivity and specificity separately, in addition to overall accuracy, to avoid misleading results due to imbalance. The details are described in the main text: in the Materials and Methods section under "Parameter Optimization" (page 4-5), and in the Results section under "Machine Learning" (page 12).
